# Supplementary material for: A Potent Antibacterial Peptide (P6) from the De Novo Transcriptome of the Microalga Aureococcus anophagefferens
Source: Int J Mol Sci. 2024 Dec 23;25(24):13736. doi: 10.3390/ijms252413736 (PMC11676368; doi:10.3390/ijms252413736)
Supplement: Supplementary file 1 [file ijms-25-13736-s001.zip › ijms-3354091-supplementary.pdf]

Figure S1: Growth inhibition of *E. coli* by four peptides. (**A**) *E. coli* growth inhibition by P2. (**B**) *E. coli* growth inhibition by P3. (**C**) *E. coli* growth inhibition by P4. (**D**) *E. coli* growth inhibition by P5.

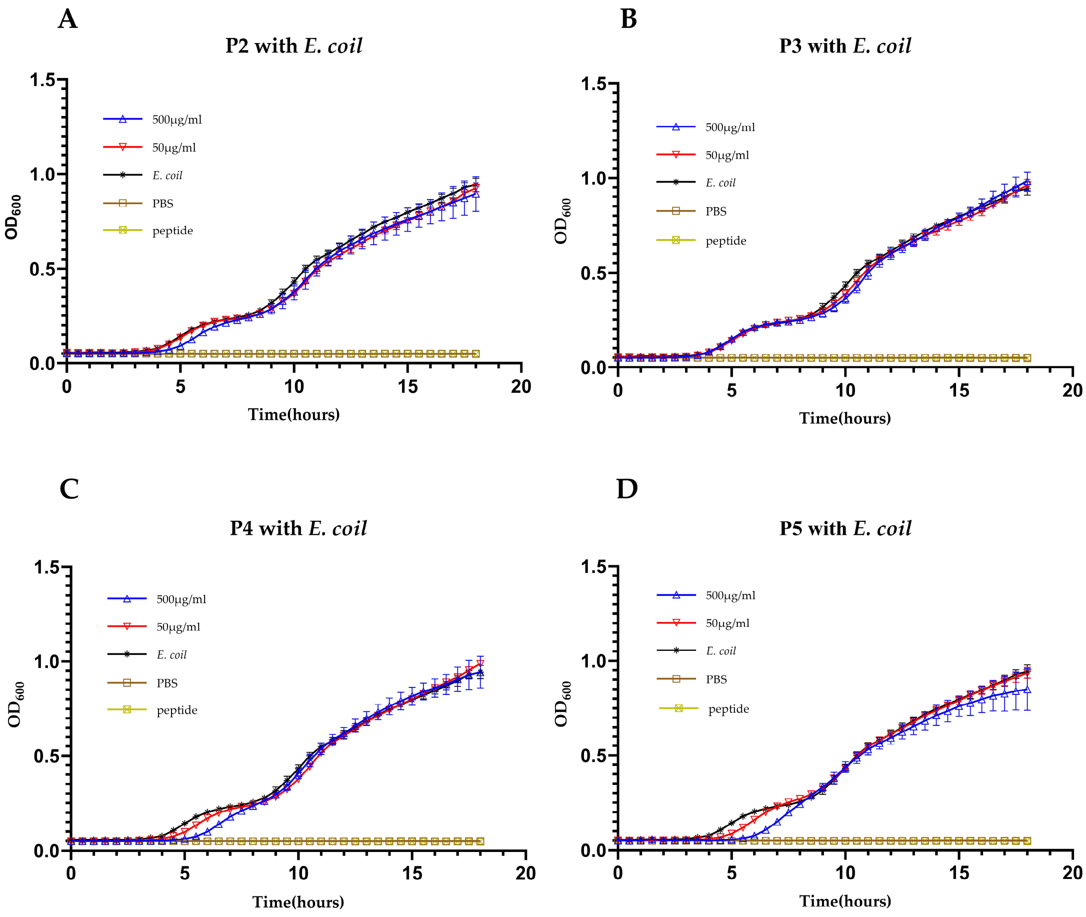

Figure S2: Growth inhibition of *S. aureus* by four peptides. (**A**) *S. aureus* growth inhibition by P2. (**B**) *S. aureus* growth inhibition by P3. (**C**) *S. aureus* growth inhibition by P4. (**D**) *S. aureus* growth inhibition by P5.

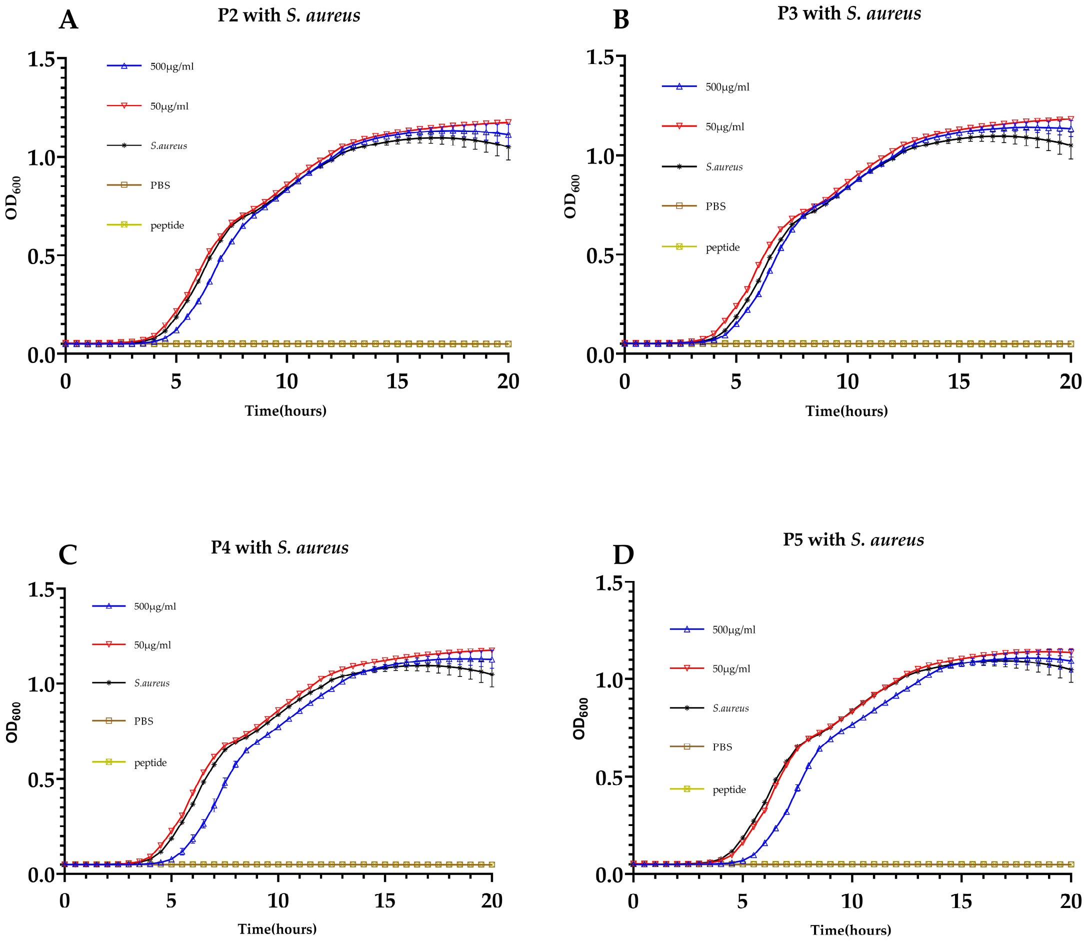

Figure S3: Growth inhibition of *M. luteus* by four peptides. (**A**) *M. luteus* growth inhibition by P2. (**B**) *M. luteus* growth inhibition by P3. (**C**) *M. luteus* growth inhibition by P4. (**D**) *M. luteus* growth inhibition by P5.

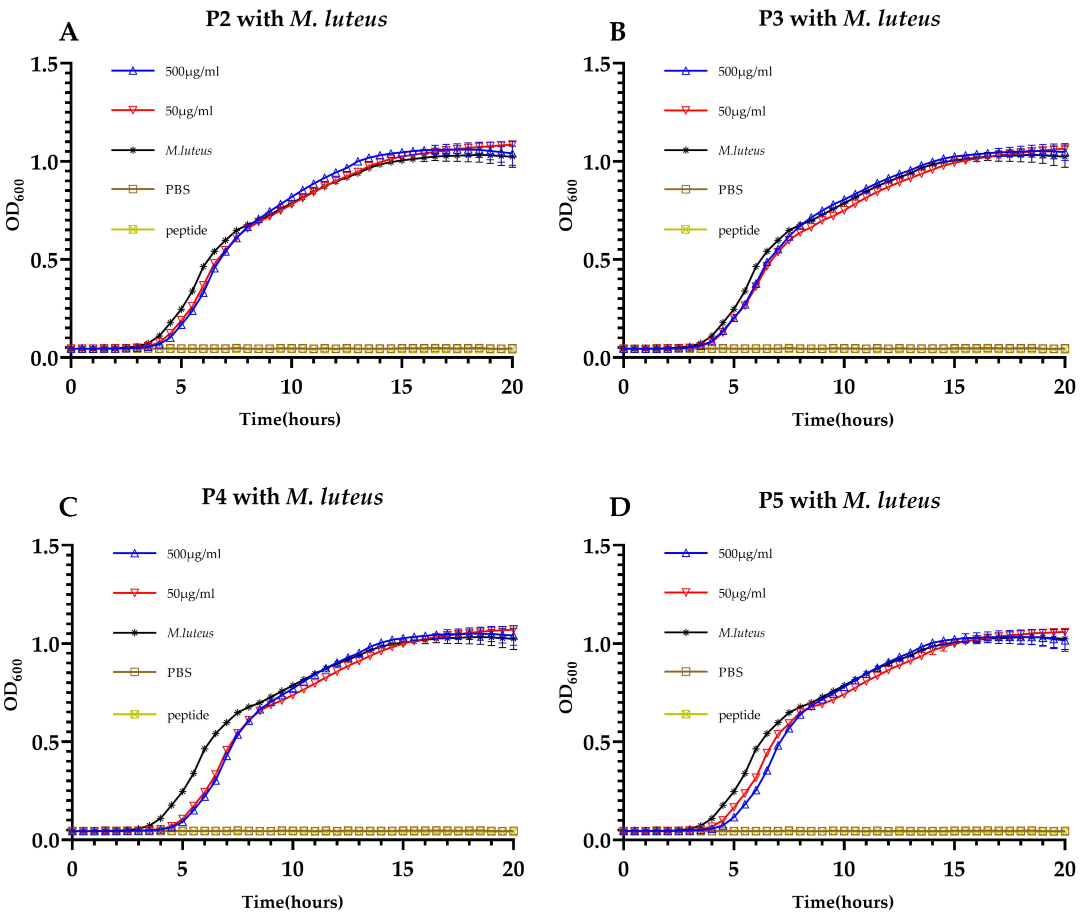

Figure S4: Growth inhibition of *P. pastoris* by four peptides. (**A**) *P. pastoris* growth inhibition by P2. (**B**) *P. pastoris* growth inhibition by P3. (**C**) *P. pastoris* growth inhibition by P4. (**D**) *P. pastoris* growth inhibition by P5.

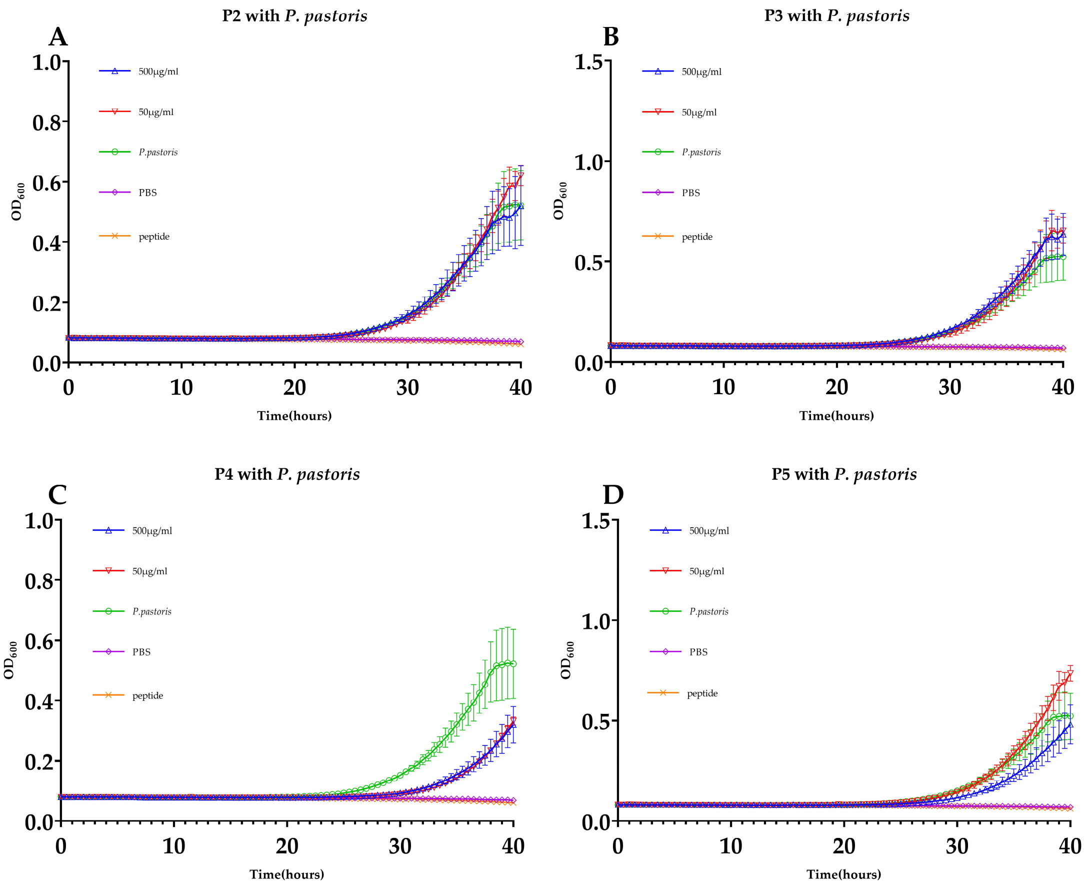

Figure S5: The hemolytic activity of synthesized AMPs at low concentrations.

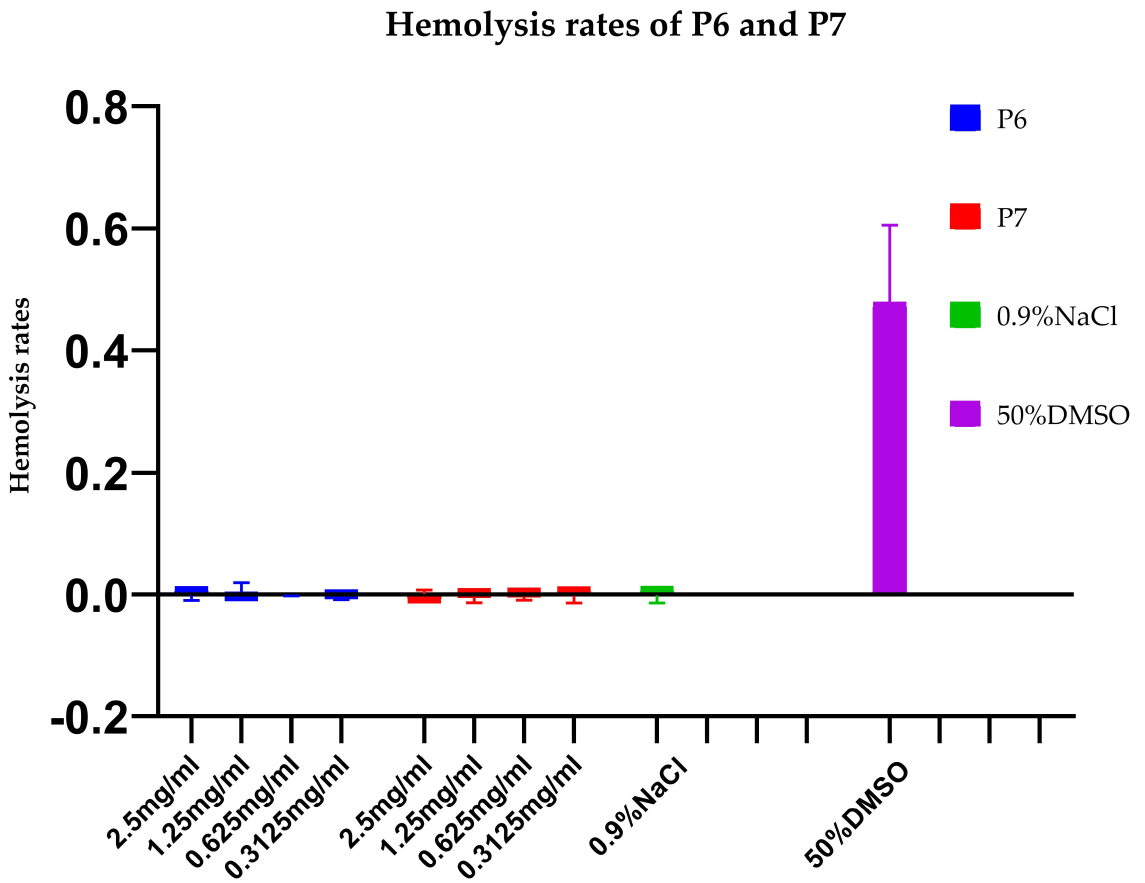

Figure S6: Structure comparison of four peptides. (**A**) The predicted 3D structures of P2. (**B**) The predicted 3D structures of P3. (**C**) The predicted 3D structures of P4. (**D**) The predicted 3D structures of P5.

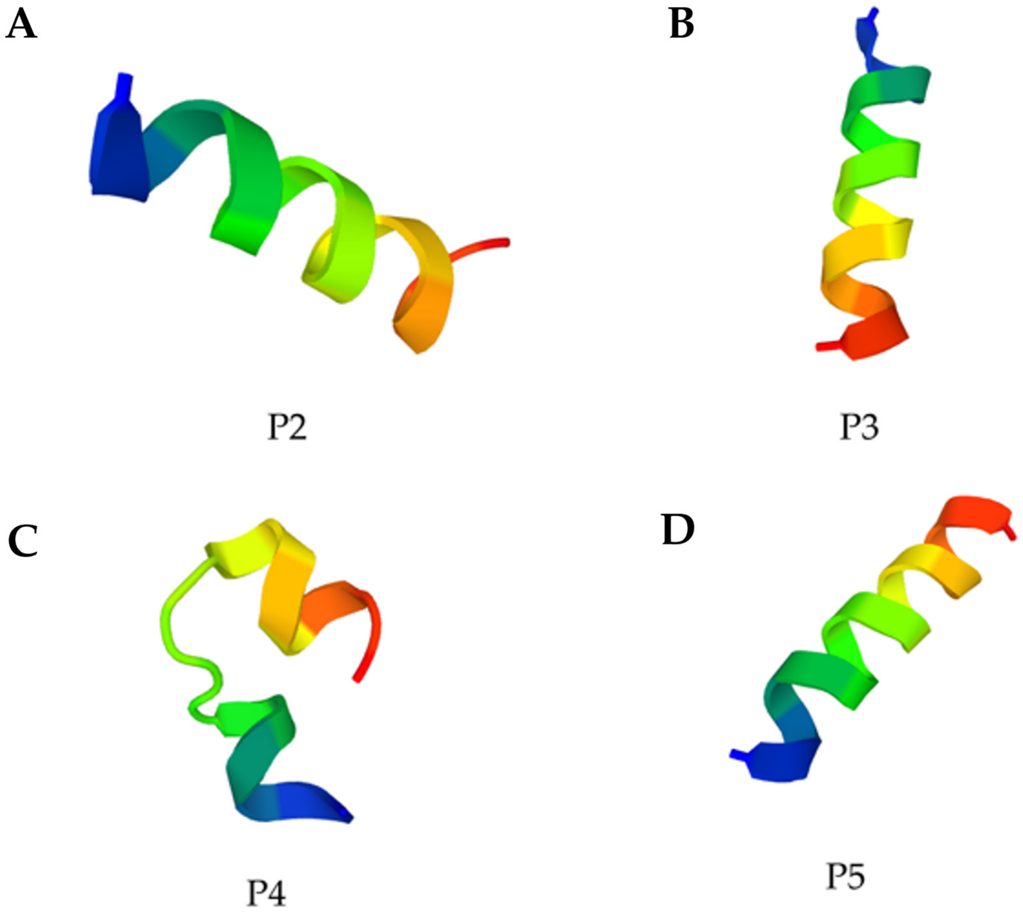

Table S1: The list of 52 N-terminal or C-terminal cleavage peptides.
